# Spore associated bacteria regulates maize root K^+^/Na^+^ ion homeostasis to promote salinity tolerance during arbuscular mycorrhizal symbiosis

**DOI:** 10.1186/s12870-018-1317-2

**Published:** 2018-06-05

**Authors:** Gopal Selvakumar, Charlotte C. Shagol, Kiyoon Kim, Seunggab Han, Tongmin Sa

**Affiliations:** 1 Department of Environmental and Biological Chemistry, College of Agriculture, Life and Environment Sciences, Chungbuk National University, Cheongju, Chungbuk 361-763 Republic of Korea; 2 Horticultural and Herbal Crop Environment Division, National Institute of Horticultural and Herbal Science, Wanju, South Korea; 3 Department of Agronomy, Benguet State University, La Trinidad, 2601 Benguet, Philippines

**Keywords:** Arbuscular mycorrhizal fungi, Spore associated bacteria, Plant-microbe interaction, *gfp*-tagging, Endophytic localization, Salt stress

## Abstract

**Background:**

The interaction between arbuscular mycorrhizal fungi (AMF) and AMF spore associated bacteria (SAB) were previously found to improve mycorrhizal symbiotic efficiency under saline stress, however, the information about the molecular basis of this interaction remain unknown. Therefore, the present study aimed to investigate the response of maize plants to co-inoculation of AMF and SAB under salinity stress.

**Results:**

The co-inoculation of AMF and SAB significantly improved plant dry weight, nutrient content of shoot and root tissues under 25 or 50 mM NaCl. Importantly, co-inoculation significantly reduced the accumulation of proline in shoots and Na^+^ in roots. Co-inoculated maize plants also exhibited high K^+^/Na^+^ ratios in roots at 25 mM NaCl concentration. Mycorrhizal colonization significantly positively altered the expression of *ZmAKT2*, *ZmSOS1,* and *ZmSKOR* genes, to maintain K^+^ and Na^+^ ion homeostasis. Confocal laser scanning microscope (CLSM) view showed that SAB were able to move and localize into inter- and intracellular spaces of maize roots and were closely associated with the spore outer hyaline layer.

**Conclusion:**

These new findings indicate that co-inoculation of AMF and SAB effectively alleviates the detrimental effects of salinity through regulation of SOS pathway gene expression and K^+^/Na^+^ homeostasis to improve maize plant growth.

**Electronic supplementary material:**

The online version of this article (10.1186/s12870-018-1317-2) contains supplementary material, which is available to authorized users.

## Background

The salinity of soil is one of the most important concerns, which are increasing progressively worldwide. More than 800 million hectares (over 6%) of the world’s total land area are affected by soil salinity (FAO 2005). Increasing salinization of arable lands adversely affects crop establishment, growth, and development contributing to huge losses in productivity [1, 2]. The high concentration of salt present in the soil causes both hyper-ionic and hyper-osmotic stress and leads to plant death [3]. Under prolonged salinity stress, the excessive Na^+^ and Cl^−^ ions are taken up by the plant cells causing toxic effects such as damage to cell organelles and plasma membrane, disruption of cell organelles, photosynthesis, and protein synthesis [4, 5]. As the majority of crop plants are glycophytes, their tolerance to salinity level beyond the threshold level reduces productivity [6]. Maize is the third most important cereal crop in the world especially in developing countries [7] and is considered as a salt sensitive cereal crop [8, 9]. In maize, Na^+^ is a major ion and under salt stress, it causes ion toxicity in plants [10].

The interaction between plant roots and salt-tolerant microorganisms helps plants alleviate the deleterious effects of salinity. Arbuscular mycorrhizal fungi (AMF) can form a mutualistic association with the roots of more than 80% of the terrestrial plants [11]. AMF have been reported to enhance plant growth under different salinity levels [5, 12–14] by enhancing the nutrient acquisition in host plants. The alleviation of salt stress by AMF has been reported through increased osmotic balance, increased activity of anti-oxidant enzymes, increased photosynthetic activity [15], increased levels of osmoregulant (proline) [16] and enhanced water uptake in plants [17]. In addition to the plant, AMF also interacts with many bacterial species in a natural environment. The interactions between AMF and bacteria have been shown to improve mutualistic fungus-host interaction [18] and plant growth [19]. Some studies have reported the positive effects of co-inoculation of AMF and plant growth promoting (PGP) bacteria on plant growth and nutrient uptake under saline stress conditions [20, 21]. Furthermore, many soil microorganisms and plant endophytic bacteria have been studied and reported to promote plant growth under various environmental conditions [22–25]. In our recent study [26], we found that AMF spore associated bacteria significantly reduced ethylene stress level and improved maize seedling growth. Co-inoculation of AMF and mycorrhizosphere bacteria increased maize plant growth by enhancing AMF hyphal length and facilitating P uptake [27]. However, the define mechanisms by which the microbes alleviate salt stress in plants remain unclear.

Due to the similar physiochemical structure of Na^+^ and K^+^, under salt stress, the excess of Na^+^ osmoticum competes for K^+^ entry into the symplast, at the transport sites. The large cytosolic Na^+^ ions compete for K^+^ binding sites and crucially restricts the metabolic activities that require K^+^. The K^+^ ion is a key component in the cytosol as it plays a critical role in protein synthesis, activation of enzymes and photosynthesis, turgor maintenance and stomatal movement [28]. AMF is known to selectively uptake K^+^ and Ca^2+^, which act as osmotic equivalents as they avoid the uptake of toxic Na^+^ [29]. However, the molecular mechanisms of regulation of uptake of K^+^ and exclusion of Na^+^ in plants by microbial inoculation remain to be elucidated. The salt overly sensitive (SOS) signaling pathway plays a significant role in maintaining ion homeostasis by regulating Na^+^ and K^+^ transport at the plasma membrane and tonoplast. The key genes responsible for ion homeostasis are *SOS1*, *SKOR*, and *AKT2* [30]. Among these, *SOS1* is widely studied for its ability to extrude Na^+^ and control xylem loading for a long-distance Na^+^ transport [31, 32]. *SKOR* is involved in the translocation of K^+^ toward shoots through xylem [33]. Further, the phloem expressing K^+^ channel, *AKT2*, is also involved in the translocation of K^+^ in shoots [34]. A previous study conducted by Estrada et al. (2013) reported that these genes are differentially regulated by AMF regulating ion homeostasis in plants under salt stress. Moreover, elucidating the gene expression regulated by endophytic bacteria might provide broad insights into the molecular mechanism involved in the alleviation of salinity stress in plants.

Therefore, this study aimed to investigate the effects of the co-inoculation of AMF and SAB on the growth of maize plant under salinity stress. The study also evaluated the association of SAB with AMF spore walls and localization in plant roots; and analyzed the alteration in the expressions of genes involved in ion homeostasis by AMF and SAB under salinity stress.

## Methods

### Strains detail

*Pseudomonas koreensis* S2CB35, a SAB, was isolated from the spore walls of AMF (Gigasporaceae) and demonstrated spore association characteristics as described earlier [35]. The isolated bacterial strain exhibited multiple plant growth-promoting characteristics, such as reduced ethylene stress and improved early growth of maize under salt stress [26]. Two AMF strains, *Gigaspora margarita* S–23 and *Claroideoglomus lamellosum* S–11, were used in the present study. They were isolated from a salt affected coastal reclamation land of Saemangeum in South Korea and propagated for the mass multiplications by a single spore mass production technique [36]. The detail of the strains and protein maker used in this study are given in Table 1.

**Table 1:** Bacterial strains and plasmids used in the present study

Plasmid/strain	Genotype or other relevant characteristics	Reference
*Escherichia coli* S17–1	Vector for plasmid pFAJ1820	Xi et al. [37]
*Escherichia coli* HB101	Helper bacteria containing pRK2013 plasmid	Xi et al. [37]
*P. koreensis* S2CB35	PGP-SAB (Genbank number: KM507143)	Selvakumar et al. [26]
*P. koreensis* S2CB35-*gfp*	*gfp*-tagged mutant representative of *P. koreensis* S2CB35	This study
*Gigaspora margarita* S-23	Propagated AMF (Genbank number: KP677599)	Selvakumar et al. [36]
*Claroideoglomus lamellosum* S-11	Propagated AMF (Genbank number: KP677595)	Selvakumar et al. [36]
pRK2013	Mobilizing plasmid	Figurski & Helinski [74]
pFAJ1820	pUT mini Tn5*gusA-pgfp*	Xi et al. [37]

### Green fluorescent protein (*gfp*)-tagging of the bacterial strain

In order to monitor the activity of SAB, the strain was tagged with GFP before inoculation. The insertion of the mini-Tn*5 gusA*::*gfp* cassette into pB10 was performed by introducing *Escherichia coli* gene Tn*5 gusA gfp* cassette (pFAJ1820) [37] into strain *P. koreensis* S2CB35 by triparental mating with the helper plasmid pRK2013 of *E. coli* HB101. The transformants were selected on a half-strength nutrient agar medium supplemented with kanamycin at 50 μg mL^− 1^. The presence of GFP in the purified transformants was confirmed by PCR amplification using following primers: YL065 (F) 5’ GCGATGTTAATGGGCAAAAA-3′ and YL066 (R) 5’-TCCATGCCATGTGTAATCCT-3′. The thermal cycling program for the amplification consisted of initial denaturation at 94 °C for 3 min, followed by 35 cycles at 94 °C for 30 s, annealing at 56 °C for 1 min, and extension at 72 °C for 1 min, with the final extension at 72 °C for 10 min [38]. The resulting amplicon of 650-bp was confirmed by gel electrophoresis. The relative fluorescence activity of *gfp*-mutant derivatives was analyzed using a flow cytometer (FACScalibur) equipped with an air-cooled argon-ion laser emitting at 488 nm (15 mW) [39]. Single *gfp*-derivative of *P. koreensis* S2CB35 was differentiated based on cell morphology, colony appearance, and growth rate characteristics of the wild type.

### Soil analysis and seedling preparation

The soil sample was collected from low salt affected reclamation land of Saemangeum, South Korea. The physicochemical properties of soil were analyzed using the standard laboratory protocols. The pH of soil was 6.0, with electrical conductivity (EC) value of 0.34 dS/m, organic matter of 5.5 g/kg, available phosphorus of 40.66 mg/kg, and with content of K, Ca, Mg, and Na measured as 0.56, 1.0, 2.2, and 0.79 cmolc/kg, respectively. The soil texture was sand 76%, silt 23.2% and clay 0.8%. The maize seeds (*Zea mays* L.) were surface sterilized using 70% ethanol for 1 min, treated for 5 min with 6% NaOCl, and washed seven times with sterile distilled water. For the bacterial treatment, the surface-sterilized seeds were imbibed in 10 mL of 0.1 M phosphate buffer (pH 6.8) containing 1 × 10^8^ cfu/mL of *P. koreensis* (S2CB35) for 4 h before the seeds were sown in a seedling tray. For the control and AMF-alone treatments, the seeds were treated with 0.1 M phosphate buffer (pH 6.8) with sterile bacteria.

### Inoculation treatments and salt stress conditions

In order to study the effects of AMF and SAB on maize growth under NaCl stress, we designed six treatments groups designated as T1 for non-treatment control and T2, T3, T4, T5, and T6 for treatments with *G. margarita*, *C. lamellosum,* SAB, *G. margarita* + SAB and *C. lamellosum* + SAB, respectively, and each one irrigated with three different concentrations of NaCl (0 mM, 25 mM, and 50 mM). The pot experiment was performed in a completely randomized block design with four replications. Each pot was filled with 2.5 kg of soil, and the mycorrhizal treatment pots received 75 g (3%) of AMF inoculum (each AMF inoculum containing approximately 200 spores and 30 root bits), which was added 1 cm below the soil surface. The control and SAB treatments received 75 g of autoclaved AMF inoculum to maintain the same nutrient content. In addition, to maintain the similar bacterial population in all of the treatments, control, and SAB treatments received 70 mL of soil extract of each AMF inoculum, which was obtained from 75 g of non-autoclaved AMF inoculum, whereas *G. margarita* inoculated pots received extracts of *C. lamellosum* and vice versa. Equally grown 7-day-old maize seedlings were transplanted into pots containing 2.5 kg of soil and maintained for 44 days after transplantation (DAT). Each plant was supplemented with 100 mL of modified Hoagland’s nutrient solution [40] regularly.

For molecular gene expression analysis and confocal laser scanning microscopy (CLSM), a separate set was prepared with the same treatments. The microbial inoculation was applied in the same ratio in 600 mL pots containing the same soil with an exception that the soil was autoclaved for 3 days consecutively to destroy all the microbes present in the soil. Both the experiments were conducted simultaneously under the same environmental conditions.

For seed bacterization, the maize seeds were soaked in 0.1 M phosphate buffer (pH 6.8) containing 1 × 10^8^ cfu/mL of SAB for 2 to 4 h. In addition to seed bacterization, 5 mL of 0.1 M phosphate buffer (pH 6.8) containing 1 × 10^8^ cfu/mL of SAB was added to the SAB and co-inoculation treatment pots at 10 and 30 DAT. The control and AMF-alone treatments received the same amount of bacterial culture with the exception that it was autoclaved at 121 °C for 15 min before inoculation. Salt stress was produced with three different NaCl concentrations (0 mM, 25 mM, and 50 mM) at 23 DAT. To avoid osmotic shock, NaCl stress was induced gradually by adding 10 mM and 15 mM to each pot after every alternate day, and the desired salt concentration was achieved after 5 days. Leaching of water from the pots was prevented by maintaining the soil water to a level below the field capacity at all the times. The maize plants were grown for another 15 days under the salt stress condition and then harvested. The soil EC value measured at the time of harvest for NaCl stress of 0 mM, 25 mM, and 50 mM were 0.49 ± 0.09 dS/m, 2.52 ± 0.35 dS/m, and 4.50 ± 0.12 dS/m, respectively. At the end of the experiment, the plants were harvested carefully, washed in distilled water, separated into leaves, shoots, and roots and were used for the analysis of different parameters.

### Determination of mineral nutrients

The biomass or dry weight of the shoots was determined after oven drying at 70 °C for at least 48 h. The proline content of the leaf was estimated according to the method described by Bates et al. [41]. The total nitrogen accumulation in the plants was measured using a Kjeldahl analyzer (K9860 Kjeldahl Analyzer, Hanon Instruments). The available phosphorus was determined using the vanadate-molybdate method, and the Ca, Mg, Na and K concentrations were estimated using inductively coupled plasma optical emission spectrometry (ICP-OES).

Chloride anions were determined in an aqueous extraction obtained from 0.4 g of dry plant material using 20 mL of deionized water. The extract was shaken for 2 h and then filtered through a Whatman number 2 filter paper and a 0.45 μm nylon membrane filter (Millipore). The diluted filtrate was then injected into an ion-exchange chromatography system (Metrohm) packed with anion separation column Metrosep A Supp 5.

### Mycorrhizal development

The root samples were washed with tap water to remove the adhering soils, and the roots were cut into the pieces of 1 cm length and stained with 0.05% trypan blue as per the method described by Phillips & Hayman [42]. The mycorrhizal root colonization (M%), colonization frequency (F%) and arbuscules abundances in the whole root system (A%) were calculated according to Trouvelot et al. [43]. The isolation of AMF spores from 50 g of soil was carried out by wet sieving and decanting method [44].

### Quantitative real time PCR

The maize root samples collected from the 600 mL pot experiment plants were washed under running water and then rinsed three times with distilled water and frozen in liquid nitrogen, ground and stored at − 80 °C. Total RNA was extracted using the RNeasy plant mini kit (Qiagen, Valencia, CA, USA) from the root samples stored at − 80 °C. cDNA was synthesized using Superscript III first strand synthesis system (Invitrogen). The gene expression analyses were carried out by quantitative reverse transcription (qRT)-PCR using CFX96 Real-Time System (Bio-Rad Laboratories, München, Germany) with SYBR Green master mix (iQ SYBR Green Supermix, Bio-Rad). Specific primers were designed by Estrada et al. [9] and used to analyze the genes: *ZmAKT2*, For (5′-CCTCAAGCATCAGGTCGAGA-3′) and *ZmAKT2*, Rev. (5′-CTCTGTAATCTTCCTGGACG-3′), *ZmSKOR*, For (5′-TCAGATCCAAGATGTCCCAG-3′) and *ZmSKOR*, Rev. (5′-TTCGTATCCTCTTAACGCAG-3′), *ZmSOS1*, For (5′-GCTTGTCACATACTTCACAG-3′) and *ZmSOS1*, Rev. (5′-ACTTGTCCACTTCACTACAC-3′). cDNAs that originated from three different biological samples were used for each gene analysis. Alpha tubulin (gi:450292) and polyubiquitin (gi:248338) were used as the internal controls for the normalization of data. All experiments were done in triplicate with three repeats.

### Preparation of plant root and spore samples for confocal microscopy

For confocal scanning laser microscopy (CLSM), the root and soil samples were used from the 600 mL pot experiment. The fresh root samples removed from SAB treated plant were washed in sterile distilled water and dried on a blotting paper. The roots were surface-sterilized and aseptically sectioned with sterile scalpel blades. The sections were mounted on a slide using fluorescence mounting medium under a coverslip. For the spores, the isolated spores were either mounted directly or after surface sterilization with 2% chloramine T and 100 μg/mL streptomycin for 30 min. The microscopic observation of root and spore samples were performed using a Leica TCS SP2 confocal system (Leica Microsystems Heidelberg GmbH) equipped with an Ar laser (gfp: excitation, 488 nm; emission filter BP, 500 to 530). Image acquisitions were performed under the objectives 20× and 40× (N.A. approximately 0.75) and were processed using the Zen lite 2012 (blue edition).

### Statistical analysis

The data were statistically analyzed using analysis of variance (ANOVA) for a completely randomized block design with SAS package 9.4 software and the differences in means were determined by the least significant differences (LSD). Duncan’s multiple-range test was performed at *P* ≤ 0.05 on each of the significant variables measured. *P* values less than 0.05 were considered as statistically significant.

## Results

### Plant growth, proline content and mycorrhizal parameters

**Fig. 1:**
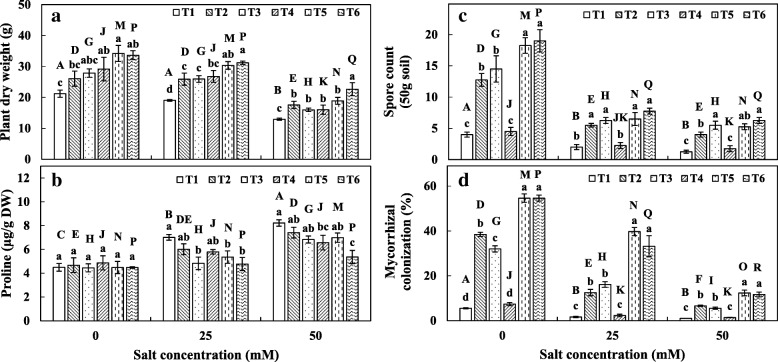
AMF and SAB co-inoculation effect on plant growth and mycorrhizal development. **a** Plant dry weight, **b** Leaf proline content, **c** AMF spore count **d** Mycorrhizal colonization. T1 – control, T2 - *Gigaspora margarita* S-23, T3 – *Claroideoglomus lamellosum* S-11, T4 – *Pseudomonas koreensis* S2CB35, T5 – T2 + T4, T6 – T3 + T4. Plants were subjected to 0 (0.5 dS/m), 25 (2.5 dS/m) or 50 mM NaCl (4.5 dS/m). Different letters indicate significant differences (*P* < 0.05) among the treatments at each salt level (a, b, c, d, e, f) or among salt levels for each treatment: T1 (A, B, C), T2 (D, E, F), T3 (G, H, I), T4 (J, K, L), T5 (M, N, O) or T6 (P, Q, R). Each value represents the mean of four replicates ± standard error (SE)

Microbial inoculation effect on maize plant growth was assessed. A significantly negative effect of salinity on growth of the plant was observed in all the treatments and a more prominent effect was evident at the highest salt concentration of 50 mM NaCl. Treatments with AMF and SAB significantly increased the dry weight of the maize as compared to the control in all salt concentration (Fig. 1a). Among the microbial treatments, co-inoculation of *C. lamellosum* with SAB significantly increased plant dry weight at 25 and 50 mM NaCl. Further, with increasing salt concentration, the corresponding increase in proline content was observed in all the treatments (Fig. 1b). However, at 0 mM NaCl, no significant differences **i**n leaf proline content were observed between treatments and control. At 25 mM NaCl, co-inoculation with AMF and SAB or *C. lamellosum* alone treatment significantly reduced the proline content. At 50 mM NaCl, co-inoculation of *C. lamellosum* with SAB and only SAB treatments significantly reduced the proline content.

The effect of salinity on mycorrhizal spore count is shown in Fig. 1c. The increase in salinity reduced AMF spore count. At 0 mM NaCl, the co-inoculation of AMF and SAB significantly increased spore count than AMF alone treatment. However, no significant differences were observed between the AMF and AMF co-inoculation with SAB treatments at 25 and 50 mM of NaCl concentration. Mycorrhizal colonization in maize roots was negatively affected by increasing salinity (Fig. 1d). A significantly high mycorrhizal colonization was observed with co-inoculation of AMF and SAB in all salt concentrations compared to AMF alone treatment. Likewise, co-inoculation of AMF and SAB exhibited significantly high colonization frequency and arbuscules abundance than AMF alone treatment (Additional file 1: Figure S1).

### Nutrient accumulation

The efficiency of nutrient uptake by plants under salt stress shows the degree of plant response to such stress. The highest salinity at 50 mM of NaCl concentration significantly lowered the nutrient uptake by plants in all the treatments. However, a significantly increased nutrient uptake in both shoot and root of maize was estimated with microbial treatments in all salt levels (Tables 2 and 3). Single and co-inoculation of AMF and SAB significantly increased total-N and phosphorous in both shoot and root at all salt levels. A significantly higher potassium uptake was observed for co-inoculation of AMF and SAB in both shoot and root tissues; however, at 50 mM of NaCl, *G. margarita* with SAB co-inoculation in shoot and *C. lamellosum* with SAB co-inoculation in root were not significantly different from control. A significantly higher calcium accumulation in shoots was observed for AMF and SAB co-inoculated plants at 0 and 50 mM NaCl. In root, co-inoculation of AMF and SAB significantly improved calcium accumulation at 0 and 50 mM NaCl. Furthermore, co-inoculation treatment showed significantly higher magnesium accumulation in shoots except at 50 mM NaCl, whereas in roots, co-inoculation treatment significantly improved magnesium accumulation at all salt levels except for *C. lamellosum* with SAB co-inoculation treatment at 50 mM NaCl.

**Table 2:** Effect of AMF and SAB co-inoculation on maize shoot nutrient accumulation under different salinity levels

Salt concentration	Treatments	Total N	P	K	Ca	Mg
		mg / plant				
0 mM	T1	819.6 ± 64.3^c, A^	68.3 ± 6.8^c, A^	470.4 ± 46.3^b, A^	40.0 ± 4.9^c, A^	32.9 ± 1.5^b, A^
	T2	1368.4 ± 247.1^bc, D^	115.3 ± 11.2^b, D^	544.5 ± 50.4^b, D^	45.9 ± 7.0^bc, DE^	41.4 ± 5.1^b, DE^
	T3	1362.9 ± 185.3^bc, G^	113.1 ± 9.9^b, G^	499.4 ± 74.1^b, G^	41.1 ± 6.5^bc, H^	42.6 ± 6.1^b, G^
	T4	1342.7 ± 101.9^bc, J^	133.0 ± 16.9^b, J^	597.2 ± 137.8^ab, J^	42.0 ± 11.9^bc, JK^	36.9 ± 7.1^b, K^
	T5	1997.7 ± 311.2^a, M^	174.4 ± 15.4^a, M^	838.0 ± 49.3^a, M^	69.4 ± 5.7^b, M^	64.3 ± 3.3^a, M^
	T6	1921.7 ± 68.9^ab, P^	180.1 ± 9.1^a, P^	817.0 ± 57.6^a, P^	96.3 ± 11.2^a, P^	69.6 ± 2.2^a, P^
25 mM	T1	808.3 ± 40.5^c, AB^	66.3 ± 1.1^d, A^	425.2 ± 11.0^d, A^	47.0 ± 5.7^b, A^	46.2 ± 2.3^b, A^
	T2	1415.6 ± 96.6^ab, D^	101.8 ± 7.8^bc, D^	470.1 ± 63.5^cd, D^	63.3 ± 8.6^ab, D^	58.7 ± 8.2^ab, D^
	T3	1218.6 ± 142.3^b, G^	98.3 ± 3.2^c, G^	566.4 ± 28.9^bcd, G^	82.1 ± 13.8^a, G^	66.9 ± 9.4^ab, G^
	T4	1441.1 ± 133.9^ab, J^	129.8 ± 10.5^b, J^	617.8 ± 44.3^abc, J^	67.3 ± 7.3^ab, J^	66.8 ± 5.8^ab, J^
	T5	1729.2 ± 117.8^a, M^	130.6 ± 5.7^b, N^	697.1 ± 77.9^ab, N^	75.4 ± 5.3^ab, M^	71.0 ± 7.0^a, M^
	T6	1765.1 ± 123.7^a, P^	161.5 ± 16.5^a, P^	757.1 ± 70.0^a, P^	87.4 ± 10.1^a, P^	78.4 ± 5.7^a, P^
50 mM	T1	553.4 ± 78.9^b, B^	50.4 ± 2.2^d, B^	295.4 ± 31.4^b, B^	30.5 ± 3.9^b, A^	42.0 ± 12.5^a, A^
	T2	768.4 ± 24.3^b, E^	67.8 ± 4.4^c, E^	287.2 ± 23.3^b, E^	31.1 ± 2.2^b, E^	31.9 ± 1.8^a, E^
	T3	764.1 ± 71.2^b, H^	72.6 ± 2.9^bc, H^	274.1 ± 18.8^b, H^	30.2 ± 1.3^b, H^	50.3 ± 17.8^a, G^
	T4	734.2 ± 98.9^b, K^	69.7 ± 5.7^c, K^	304.6 ± 20.7^b, K^	29.8 ± 4.2^b, K^	33.2 ± 4.6^a, K^
	T5	1066.9 ± 21.5^a, M^	87.5 ± 4.1^b, O^	352.3 ± 12.2^b, O^	50.6 ± 3.7^a, N^	46.1 ± 1.6^a, N^
	T6	1265.4 ± 170.4^a, Q^	110.5 ± 11.6^a, Q^	467.4 ± 40.8^a, Q^	63.4 ± 10.8^a, P^	60.7 ± 8.6^a, P^

T1 – control, T2 - *Gigaspora margarita* S-23, T3 – *Claroideoglomus lamellosum* S-11, T4 – *Pseudomonas koreensis* S2CB35, T5 – T2 + T4, T6 – T3 + T4. Plants were subjected to 0 (0.5 dS/m), 25 (2.5 dS/m) or 50 mM NaCl (4.5 dS/m). Different letters indicate significant differences (*P* < 0.05) among the treatments at each salt level (a, b, c, d, e, f) or among salt levels for each treatment: T1 (A, B, C), T2 (D, E, F), T3 (G, H, I), T4 (J, K, L), T5 (M, N, O) or T6 (P, Q, R). Each value represents the mean of four replicates ± standard error (SE)

**Table 3:** Effect of AMF and SAB co-inoculation on maize root nutrient accumulation under different salinity levels

Salt concentration	Treatments	Total N	P	K	Ca	Mg
		mg / plant				
0 mM	T1	78.8 ± 2.8^d, A^	13.9 ± 0.9^e, A^	51.5 ± 5.3^b, A^	5.6 ± 0.7^c, AB^	4.2 ± 0.3^d, AB^
	T2	165.7 ± 26.1^cd, D^	22.1 ± 3.1^cd, D^	53.5 ± 7.4^b, D^	9.7 ± 0.7^c, E^	6.4 ± 0.2^cd, E^
	T3	250.4 ± 39.6^bc, G^	19.2 ± 3.2d^e, G^	80.3 ± 17.0^ab, G^	17.0 ± 1.7^b, G^	8.9 ± 0.8^bc, G^
	T4	196.1 ± 18.8^bcd, J^	36.7 ± 0.9^a, J^	106.1 ± 18.6^a, J^	20.9 ± 2.6^ab, J^	11.2 ± 1.2^b, J^
	T5	303.6 ± 27.5^ab, M^	28.6 ± 2.3^bc, M^	77.8 ± 17.9^ab, M^	21.2 ± 2.2^ab, M^	9.9 ± 1.2^b, MN^
	T6	384.7 ± 80.7^a, P^	30.3 ± 3.0^ab, P^	107.4 ± 17.3^a, P^	28.1 ± 4.2^a, P^	16.5 ± 1.4^a, P^
25 mM	T1	83.6 ± 4.7^c, A^	7.2 ± 0.5^d, B^	12.4 ± 3.8^c, B^	6.9 ± 1.9^a, A^	5.3 ± 1.8^b, A^
	T2	213.6 ± 42.1^b, D^	14.4 ± 1.0^c, E^	34.1 ± 3.7^b, E^	14.7 ± 1.3^a, D^	11.5 ± 1.3^a, D^
	T3	206.9 ± 47.5^b, G^	16.2 ± 3.3^bc, GH^	21.9 ± 4.5^bc, H^	25.0 ± 13.3^a, G^	8.8 ± 0.8^ab, G^
	T4	183.9 ± 14.8^b, JK^	20.1 ± 1.8^bc, K^	33.6 ± 8.7^b, K^	6.7 ± 1.4^a, K^	5.6 ± 1.0^b, K^
	T5	341.3 ± 33.3^a, M^	28.1 ± 2.9^a, M^	60.1 ± 10.0^a, M^	18.9 ± 1.3^a, MN^	11.3 ± 1.3^a, M^
	T6	275.5 ± 20.3^ab, PQ^	22.4 ± 1.9^ab, P^	32.0 ± 2.9^b, Q^	12.5 ± 1.9^a, Q^	5.2 ± 1.2^b, Q^
50 mM	T1	62.8 ± 3.0^b, B^	4.6 ± 0.7^c, C^	5.3 ± 0.9^b, B^	2.7 ± 0.3^c, B^	1.4 ± 0.2^b, B^
	T2	139.2 ± 21.9^a, D^	10.5 ± 1.3^ab, E^	13.7 ± 2.1^ab, F^	5.4 ± 1.1^c, F^	2.4 ± 0.5^b, F^
	T3	127.3 ± 30.3^a, G^	8.3 ± 0.8^bc, H^	9.5 ± 2.2^b, H^	3.5 ± 0.8^c, G^	1.8 ± 0.5^b, H^
	T4	139.5 ± 19.9^a, K^	10.9 ± 1.5^ab, L^	18.5 ± 4.9^ab, K^	4.5 ± 0.6^c, K^	3.0 ± 0.5^b, K^
	T5	173.1 ± 22.0^a, N^	14.0 ± 1.6^a, N^	24.5 ± 7.0^a, N^	13.6 ± 1.9^a, N^	7.2 ± 2.2^a, N^
	T6	134.8 ± 12.6^a, Q^	11.8 ± 1.4^ab, Q^	16.3 ± 7.3^ab, Q^	10.0 ± 1.1^b, Q^	3.5 ± 0.6^a, Q^

T1 – control, T2 - *Gigaspora margarita* S-23, T3 – *Claroideoglomus lamellosum* S-11, T4 – *Pseudomonas koreensis* S2CB35, T5 – T2 + T4, T6 – T3 + T4. Plants were subjected to 0 (0.5 dS/m), 25 (2.5 dS/m) or 50 mM NaCl (4.5 dS/m). Different letters indicate significant differences (*P* < 0.05) among the treatments at each salt level (a, b, c, d, e, f) or among salt levels for each treatment: T1 (A, B, C), T2 (D, E, F), T3 (G, H, I), T4 (J, K, L), T5 (M, N, O) or T6 (P, Q, R). Each value represents the mean of four replicates ± standard error (SE)

### Sodium and chloride uptake

**Fig. 2:**
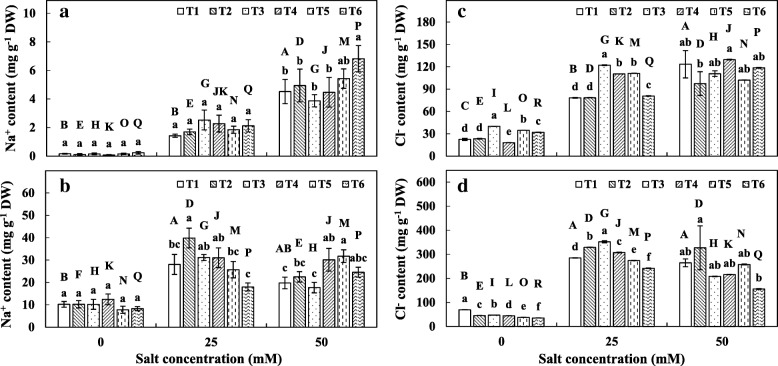
Sodium (Na^+^) and Chloride (Cl^−^) content in maize plants. **a** Na^+^ content in shoot, **b** Na^+^ content in root, **c** Cl^−^ content in shoot **d** Cl^−^ content in root. See legend for Fig. 1. Each value represents the mean of four replicates (Na^+^) or three replicates (Cl^−^) ± standard error (SE)

Under salt stress, plants take up more sodium ion than potassium. A significant increase in sodium accumulation in maize shoots was observed with the increase in salinity (Fig. 2a). At 0 and 25 mM NaCl, no significant difference was observed between the treatments and control. However, at 50 mM NaCl, the co-inoculation of *C. lamellosum* with SAB significantly enhanced sodium accumulation in a shoot. In root tissues, the sodium accumulation was higher at 25 and 50 mM NaCl compared to 0 mM NaCl (Fig. 2b). However, no significant differences were observed between 25 and 50 mM NaCl in control plants. At 25 mM NaCl, only the co-inoculation of *C. lamellosum* with SAB showed a significantly reduced sodium accumulation in root tissues than all other treatments.

The accumulation of chloride ions was significantly increased in maize shoot tissues with the increase in salinity (Fig. 2c). In shoot tissues, at 0 and 25 mM NaCl, all the treatments showed an increased chloride accumulation except with *G. margarita*. However, at 50 mM NaCl, no significant differences were observed among the treatments. In contrast, root tissues exhibited lower chloride accumulation than control at 0 mM NaCl in all the treatments (Fig. 2d). At 25 mM NaCl, a single inoculation of AMF and SAB enhanced accumulation of chloride ions than control and the co-inoculation treatments showed lower chloride accumulation than control. However, at 50 mM NaCl, only co-inoculation of *C. lamellosum* with SAB showed lower chloride accumulation, other treatments exhibited no significant differences as compared to control.

### K^+^/Na^+^ ratios

**Fig. 3:**
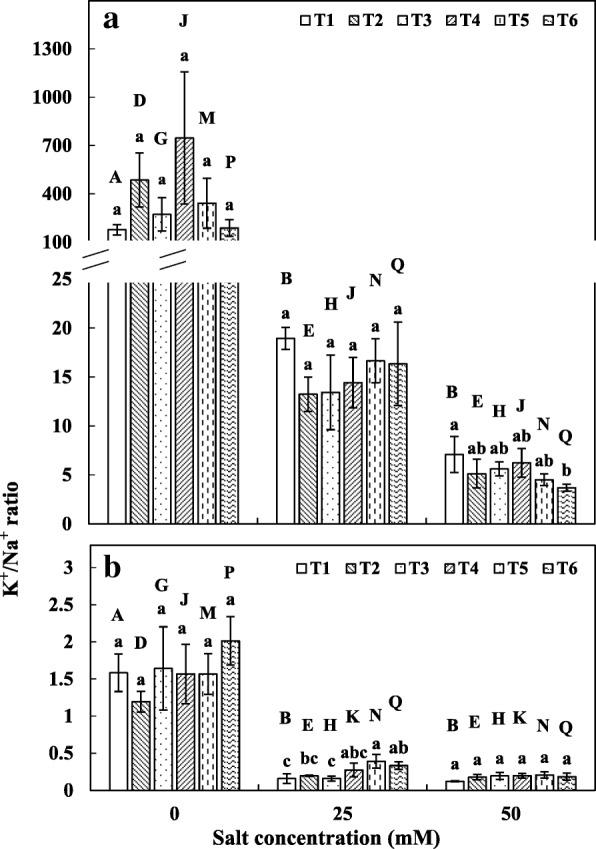
K^+^/Na^+^ ratio in maize plants. **a** K^+^/Na^+^ ratio in shoot, **b** K^+^/Na^+^ ratio in root. See legend for Fig. 1

In both shoots and roots of maize, the K^+^/Na^+^ ratio was negatively affected by salinity at all the concentration. The effect was more prominent in shoots, where the differences between non-saline treatment and either of the salt treatments were highly significant (Fig. 3a). However, microbial treatments did not show significant differences from control at 0 and 25 mM NaCl. At 50 mM NaCl, only the co-inoculation of *C. lamellosum* with SAB showed lower K^+^/Na^+^ ratio. In root tissues, at 0 and 50 mM NaCl, no differences were observed between the treatments, whereas at 25 mM NaCl, co-inoculation treatments showed significantly higher K^+^/Na^+^ ratio (Fig. 3b).

### Ion transporter gene expression analysis

**Fig. 4:**
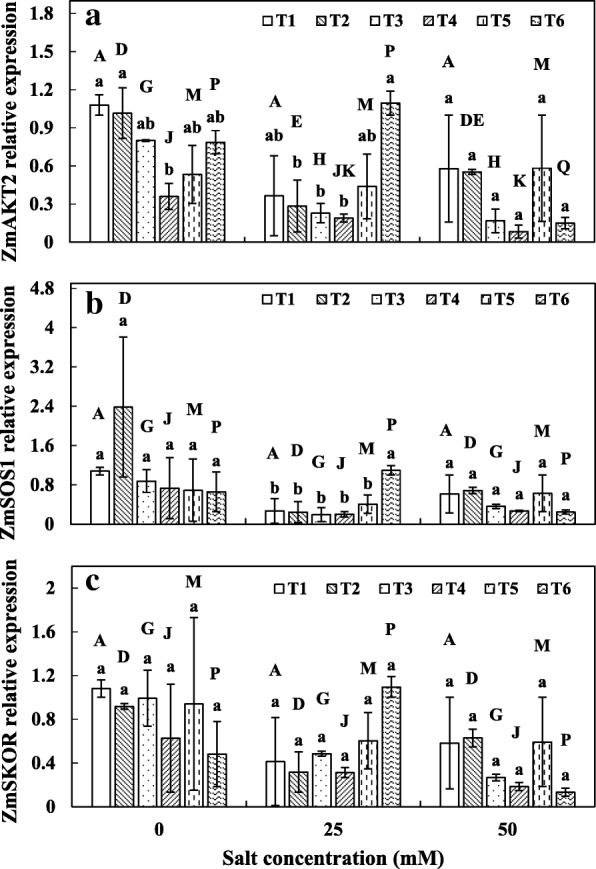
Gene expression analysis in maize roots by real-time quantitative PCR. **a** *ZmAKT2*, **b** *ZmSOS1*, **c** *ZmSKOR*. See legend for Fig. 1

Ion analysis suggest that microbial colonization affect tissue K^+^ and Na^+^. We therefore tested whether SOS genes expression are regulated by AMF and SAB colonization. AMF colonization significantly altered the K^+^ and Na^+^ accumulation in plants. We have tested the membrane transporters responsible for K^+^ uptake and translocation along with Na^+^ deposition. Our result showed that the expression of *ZmAKT2* gene was differentially affected by single and co-inoculation of AMF and SAB with increasing salinity (Fig. 4a). At 0 mM NaCl, plants inoculated with SAB alone showed significantly lower gene expression compared to control and other microbial treatments. At 25 and 50 mM NaCl, no significant differences were observed between the treatments. When compared to the salt concentrations, only plants treated with the co-inoculation of *C. lamellosum* and SAB exhibited increased gene expression (39%) at 25 mM NaCl from 0 mM NaCl; however, the expression was reduced significantly at 50 mM NaCl.

The expression of *ZmSOS1* and *ZmSKOR* were negatively affected by salinity (Fig. 4b, c). No significant difference in the gene expression of both the genes was observed among different salt concentrations. Each treatment exhibited different gene expression at all salt concentration for both *ZmSOS1* and *ZmSKOR* genes. Only plants treated with co-inoculation of *C. lamellosum* and SAB showed significantly higher expression at 25 mM NaCl for *ZmSOS1*. The higher expression of *ZmSKOR* was observed in plants treated with co-inoculation of *C. lamellosum* and SAB at 25 mM NaCl compared to 0 mM NaCl.

### Confocal scanning microscopy

**Fig. 5:**
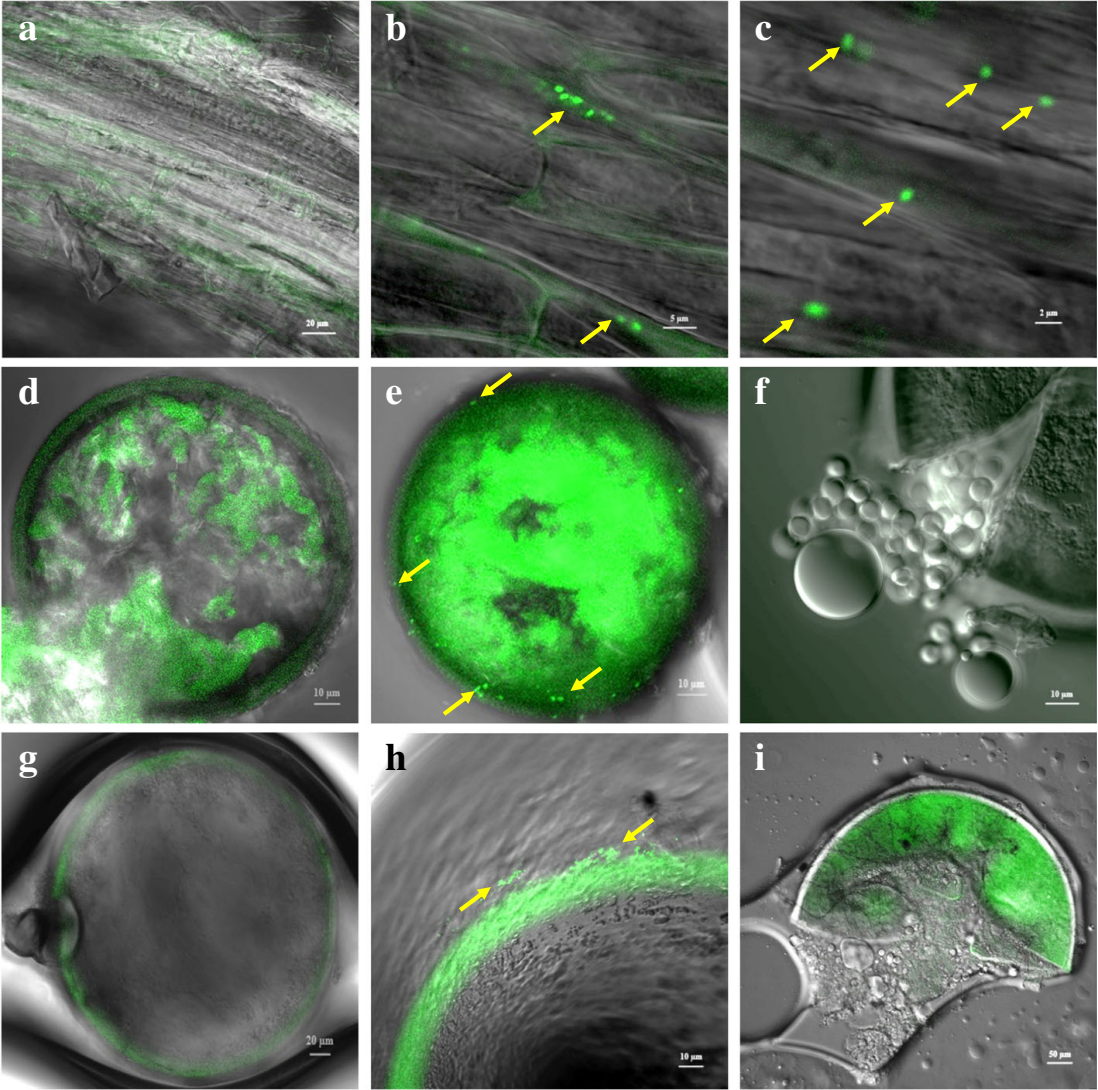
SAB *Pseudomonas koreensis* S2CB35-*gfp* colonization in maize plant roots and association on AMF spore walls. **a** – control, **b** – intercellular colonization of SAB, **c** – Intra cellular colonization of SAB. **d**, **e**, **f** - *Claroideoglomus lamellosum* S-11, **g**, **h**, **i** - *Gigaspora margarita* S-23. **d** and **g** – Control, **e** and **h** – SAB colonization on AMF surface, **f** and **i** – No endosporic association. Arrow indicates the *gfp*-tagged SAB

The roots of harvested maize plants were observed under CLSM to confirm the localization of the *gfp*-tagged SAB strain, *P. koreensis*. Fluorescent bacterial cells were observed to be absent in uninoculated control plants (Fig. 5a). However, plants inoculated with *gfp*-tagged SAB showed that the fluorescent bacterial cells were localized on the surface of the roots (Additional file 2: Figure S2). Several SAB were also able to move and colonize to inter and intracellular spaces (Fig. 5b and c). SAB *P. koreensis* S2CB35 efficiently colonized the rhizoplane, moved into root tissues, and localized themselves to intercellular spaces of root tissues. Furthermore, the ability of SAB to associate with the spore walls were also observed (Fig. 5d-i). No SAB colonization was observed on the spore walls of AMF isolated from pots treated with *C. lamellosum* or *G. margarita* alone (Fig. 5d, g). Clear fluorescent bacterial cells were observed on the spore walls of AMF isolated from co-inoculation of AMF and SAB treatment pots (Fig. 5e, h, Additional file 3: Figure S3 and Additional file 4: Figure S4). However, surface sterilized and the broken spores exhibited no endosporic colonization of SAB (Fig. 5f, i) suggesting that the SAB was limited to the outer surface of AMF spore walls.

## Discussion

Plant-microbe symbiosis is an important component for plant’s ability to cope with the adverse environmental conditions. Previous studies have demonstrated important mechanisms employed by AMF to promote plant growth under salinity stress [17, 45]. However, these experiments were based on the inoculation of AMF alone. In a recent report by Berta et al. [46], it was demonstrated that the co-inoculation of AMF and soil rhizobia markedly promoted the growth of maize plant in field conditions than as a single inoculation. Although mycorrhizal colonization is considered nonspecific, it can be enhanced by co-inoculation with mycorrhizal helper bacteria [19, 27]. In the present work, we analyzed the significance of application of two indigenous AMF isolates with a bacterium isolated from the surface of AMF spore walls on maize. It has been reported that salinity negatively affects the plant growth and development [47, 48]. Several studies have reported that salinity reduced growth, leaf area, chlorophyll content, nutrient uptake and photosynthesis [15, 49, 50]. In this study, dry weight of maize plant decreased with the increase in salinity. However, the co-inoculation of AMF and SAB significantly increased plant dry weight under salinity stress. Our results indicate that under salinity, microbial inoculation plays a significant role in promoting plant growth.

Proline is an important osmoprotectant osmolyte and is known to play a vital role in protecting plants from various environmental stresses [51]. Our results demonstrate that under salinity stress, maize plants accumulated a higher amount of proline. However, co-inoculation of AMF and SAB significantly reduced proline accumulation in plants under salinity stress. Previous reports also suggested that microbial inoculation decreased the proline accumulation in plants [16, 40] under stressful environment. Mycorrhizal colonization was reported to reduce under salinity [52]. Similarly, in the present study, mycorrhizal colonization was reduced under salt stress; however, the co-inoculation of SAB with AMF increased mycorrhizal colonization in all the salt concentration than AMF treatment alone. Our results are in accordance with Hashem et al. [53], they reported that the co-inoculation of AMF with endophytic bacteria increased the mycorrhizal colonization in *Acacia gerrardii* under salt stress. Although mycorrhizal helper bacteria is known to improve fungal growth and colonization efficiency, we found SAB had no positive influence on spore production under salinity stress.

Soil salinity affects the nutrient uptake by plants and transport to shoots [54]. Our results indicate that salinity decreased the nutrient uptake by plants. Nitrogen is an essential constituent of plant chlorophyll, amino acids, and in the energy transfer compound of ATP (adenosine triphosphate). Increased salinity reduced the uptake of nitrogen; however, inoculation/co-inoculation treatments significantly increased the nitrogen uptake by plants, in the present study. The phosphate (P) solubilizing microorganisms (PSM) are capable of transforming insoluble P into a plant accessible soluble form. AMF is well known for their capability to enhance P uptake by plants. Further, PSM have been reported to increase P uptake by a plant [55]. An increased P uptake by plants was observed in our study with the use of P solubilizing SAB *P. koreensis* S2CB35; nevertheless, no difference was observed between AMF alone treatment and SAB alone treatment. However, a higher P accumulation in plants treated with co-inoculation suggests that the plants might have benefited from both AMF and SAB. A similar study by Battini et al., [27] also reported that co-inoculation of AMF and SAB significantly increased maize plant growth by facilitating the P uptake.

Furthermore, plants also accumulate inorganic solutes such as potassium to maintain osmotic or the turgor pressure in addition to organic solute like proline [56] under salinity stress. A higher level of Na^+^ ions present in the soil competes with K^+^ ions resulting in an increased accumulation of Na^+^ ions in plants [57]. K^+^ is required for the osmotic balance, has a role in the opening and closing of the stoma, and is an essential factor in protein biosynthesis. Giri et al. [58] reported that these functions of K^+^ cannot be substituted with Na^+^ ions accumulated in the cytosol. In this study, the co-inoculation enhanced the accumulation of K^+^ in both root and shoot under salinity stress. According to Estrada et al. [9], root tissues have a higher accumulation of Na^+^ than shoots. Cantrell & Linderman [59] reported that the accumulated Na^+^ in mycorrhizal roots may compartmentalize in cell vacuoles and in AMF hyphae to prevent translocation to the shoots. The co-inoculation treatments at 25 mM NaCl and *C. lamellosum* alone treatment at 50 mM NaCl showed a lower Na^+^ accumulation in roots. High ratios of K^+^/Na^+^ were found in our study, suggesting that microbial treatments had a significant impact on K^+^/Na^+^ ratio than non-inoculated plant roots under salinity stress. It has been suggested that the maintenance of high K^+^/Na^+^ ratios in shoots of glycophytes is an important mechanism to cope with the salinity stress [60]. In contrast to previous reports [9, 29], our result indicates that microbial treatments inhibited Cl^−^ uptake by plants. Co-inoculation treatments exhibited lower Cl^−^ uptake by plant roots under salinity stress. A recent study by Elhindi et al. [61] demonstrated that mycorrhizal treated plants showed lower Cl^−^ accumulation. Although, a slight increase in Ca^2+^ and Mg^2+^ was recorded at 25 mM NaCl. The increase in salinity reduced the accumulation of these ions. The negative impact of soil salinity on Ca^2+^ and Mg^2+^ uptake was also reported earlier [47, 52], which is in concordance with our findings.

Previous reports showed that the inoculation of symbiotic microbes improves salt tolerance in plants by improving nutrient uptake [62], antioxidant activity [63], and increased synthesis of photosynthetic pigments [53]. Moreover, ion homeostasis is maintained by plants to resist salinity stress. It has also been reported that Na^+^/H^+^ antiporter overexpression affects both salinity tolerance and K^+^ nutrition [64]. *AKT* belongs to the family of plant K^+^ inward channel and is responsible for the uptake of K^+^. *AKT2* plays a role in sugar loading of the phloem in long distance transport [65]. On the other hand, the *SKOR* channel influences the xylem loading of K^+^ [30]. Our results showed that different treatments had different effects on expression of these genes. A highly significant difference was observed at 25 mM NaCl where the plants treated with co-inoculation of *C. lamellosum* and SAB considerably increased the expression of AKT and SKOR. The Na^+^ antiporter *SOS1* has been shown to be involved in the extrusion of Na^+^ [32]. We found a higher expression of *ZmSOS1* gene at 25 mM NaCl in plants co-inoculated with *C. lamellosum* and *P. koreensis* S2CB35 which correlates with the low Na^+^ content in the root tissues. Mmycorrhizal treated plants showed a considerably higher gene expression than non-inoculated plants and SAB alone.

SAB *P. koreensis* S2CB35 was able to effectively colonize maize root tissues and migrate to inter- and intracellular spaces of root cells. Kost et al. [66] also found that bacteria by utilizing key constituents malate and oxalate of root exudates as sole carbon source were able to effectively colonize the root surfaces. The strain used in the present study was able to utilize malate as a sole carbon source; however, it was not able to utilize oxalate. The bacterial species, *P. koreensis* was initially isolated from farming soils in Korea [67]. *P. koreensis* was also reported to exist in various environmental conditions such as in extreme oligotrophic sites [68], plant endophytes [69], and heavy metal contaminated sites [25]. In the present study, the strain *P. koreensis* S2CB35 was isolated from the surface of AMF spores. CLSM view of AMF spore showed that *gfp*-tagged SAB was effectively associated with spore walls of both the AMF strains. The localization of bacteria on spore have previous been studied [70, 71] and reported to have positive effect on AMF germination. In addition, diverse bacterial communities were identified to be associated with AMF spores and shown to have multifunctionality [72, 73].

## Conclusions

In conclusion, our study indicates that co-inoculation of AMF and SAB improved the growth and salt tolerance of maize. Mycorrhizal and bacterial treatments increased nutrient uptake by plants and increased ratios of K^+^/Na^+^ in root and shoot tissues under salinity stress. A significant positive alteration in gene expression of ion homeostasis genes was demonstrated by mycorrhizal treatments. Co-inoculation of AMF and SAB exhibited an improved capability to alleviate inhibitory effects of salinity than AMF or SAB alone treatments. SAB was found to be associated with the spore walls of AMF and was localized in inter- and intra-cellular spaces of maize roots. These results highlight the importance of considering co-inoculation to effectively alleviate detrimental effects of salinity and improve plant growth in salinized soils. Further, the understanding of molecular mechanisms involved in the association between AMF and bacteria are likely to provide benefits to the use of effective microbial consortium in sustainable agricultural practices.

## Additional files

Additional file 1: **Figure S1.** AMF and SAB co-inoculation effect on mycorrhizal colonization frequency and arbuscules abundance. (A) Colonization frequency (B) Arbuscules abundance. T1 – control, T2 - *Gigaspora margarita* S-23, T3 – *Claroideoglomus lamellosum* S-11, T4 – *Pseudomonas koreensis* S2CB35, T5 – T2 + T4, T6 – T3 + T4. Plants were subjected to 0 (0.5 dS/m), 25 (2.5 dS/m) or 50 mM NaCl (4.5 dS/m). Different letters indicate significant differences (*P* < 0.05) among the treatments at each salt level (a, b, c, d, e, f) or among salt levels for each treatment: T1 (A, B, C), T2 (D, E, F), T3 (G, H, I), T4 (J, K, L), T5 (M, N, O) or T6 (P, Q, R). Each value represents the mean of four replicates ± standard error (SE). (TIF 5431 kb)

Additional file 2: **Figure S2.** SAB *Pseudomonas koreensis* S2CB35-*gfp* colonization in maize plant roots. Arrow indicates the *gfp*-tagged SAB. (TIF 15878 kb)

Additional file 3: **Figure S3.** SAB *Pseudomonas koreensis* S2CB35-*gfp* colonization on *Claroideoglomus lamellosum* S-11. Arrow indicates the *gfp*-tagged SAB. (TIF 14024 kb)

Additional file 4: **Figure S4.** SAB *Pseudomonas koreensis* S2CB35-*gfp* colonization on *Gigaspora margarita* S-23. Arrow indicates the *gfp*-tagged SAB. (TIF 9889 kb)

## References

[1] Wang W, Vinocur B, Altman A (2003). Plant responses to drought, salinity and extreme temperatures: towards genetic engineering for stress tolerance. Planta.

[2] Mathur N, Singh J, Bohra S, Vyas A (2007). Arbuscular mycorrhizal status of medicinal halophytes in saline areas of Indian Thar Desert. Int J Soil Sci.

[3] Porcel R, Aroca R, Ruiz-Lozano JM (2012). Salinity stress alleviation using arbuscular mycorrhizal fungi. A review. Agron Sustain Dev.

[4] Juniper S, Abbott L (1993). Vesicular-arbuscular mycorrhizas and soil salinity. Mycorrhiza.

[5] Feng G, Zhang FS, Li XL, Tian CY, Tang C, Rengel Z (2002). Improved tolerance of maize plants to salt stress by arbuscular mycorrhiza is related to higher accumulation of soluble sugars in roots. Mycorrhiza.

[6] Khan MA, Shirazi MU, Ali M, Mumtaz S, Sherin A, Ashraf MY (2006). Comparative performance of some wheat genotypes growing under saline water. Pak J Bot.

[7] Maize MD (2003). Post harvest operations.

[8] Maas EV, Hoffman GJ (1977). Crop salt tolerance - current assessment. J Irrig Drain Div.

[9] Estrada B, Aroca R, Maathuis FJM, Barea JM, Ruiz-Lozano JM (2013). Arbuscular mycorrhizal fungi native from a Mediterranean saline area enhance maize tolerance to salinity through improved ion homeostasis. Plant Cell Environ.

[10] Fortmeier R, Schubert S (1995). Salt tolerance of maize (*Zea mays* L): the role of sodium exclusion. Plant Cell Environ.

[11] Smith SE, Read DJ (2010). Mycorrhizal symbiosis.

[12] Ruiz-Lozano JM, Azcon R, Gomez M (1996). Alleviation of salt stress by arbuscular-mycorrhizal *Glomus* species in *Lacuca sativa* plants. Physiol Plant.

[13] Tian CY, Feng G, Li XL, Zhang FS (2004). Different effects of arbuscular mycorrhizal fungal isolates from saline or non-saline soil on salinity tolerance of plants. Appl Soil Ecol.

[14] Sannazzaro AI, Ruiz OA, Albertó EO, Menéndez AB (2006). Alleviation of salt stress in *Lotus glaber* by *Glomus intraradices*. Plant Soil.

[15] Sheng M, Tang M, Chen H, Yang B, Zhang F, Huang Y (2008). Influence of arbuscular mycorrhizae on photosynthesis and water status of maize plants under salt stress. Mycorrhiza.

[16] Jahromi F, Aroca R, Porcel R, Ruiz-Lozano JM (2008). Influence of salinity on the in vitro development of *Glomus intraradices* and on the in vivo physiological and molecular responses of mycorrhizal lettuce plants. Microb Ecol.

[17] Çekiç FÖ, Ünyayar S, Ortaş I (2012). Effects of arbuscular mycorrhizal inoculation on biochemical parameters in Capsicum annuum grown under long term salt stress. Turk J Bot.

[18] Artursson V, Finlay RD, Jansson JK (2006). Interactions between arbuscular mycorrhizal fungi and bacteria and their potential for stimulating plant growth. Environ Microbiol.

[19] Gamalero E, Berta G, Massa N, Glick BR, Lingua G (2008). Synergistic interactions between the ACC deaminase-producing bacterium *Pseudomonas putida* UW4 and the AM fungus *Gigaspora rosea* positively affect cucumber plant growth. FEMS Microbiol Ecol.

[20] Rabie GH, Aboul-Nasr MB, Al-Humiany A (2005). Increased salinity tolerance of cowpea plants by dual inoculation of an arbuscular mycorrhizal fungus *Glomus clarum* and a nitrogen-fixer *Azospirillum brasilense*. Mycobiology.

[21] Lee Y, Krishnamoorthy R, Selvakumar G, Kim K, Sa T (2015). Alleviation of salt stress in maize plant by co-inoculation of arbuscular mycorrhizal fungi and *Methylobacterium oryzae* CBMB20. J Korean Soc Appl Biol Chem.

[22] Jalili F, Khavazi K, Pazira E, Nejati A, Rahmani HA, Sadaghiani HR (2009). Isolation and characterization of ACC deaminase-producing fluorescent pseudomonads, to alleviate salinity stress on canola (*Brassica napus* L.) growth. J Plant Physiol.

[23] Siddikee MA, Glick BR, Chauhan PS, jong YW, Sa T (2011). Enhancement of growth and salt tolerance of red pepper seedlings (*Capsicum annuum* L.) by regulating stress ethylene synthesis with halotolerant bacteria containing 1-aminocyclopropane-1-carboxylic acid deaminase activity. Plant Physiol Biochem.

[24] Mahdhi M, Fterich A, Rejili M, Rodriguez-Llorente ID, Mars M (2012). Legume-nodulating bacteria (LNB) from three pasture legumes (*Vicia sativa*, *Trigonella maritima* and *Hedysarum spinosissimum*) in Tunisia. Ann Microbiol.

[25] Babu AG, Shea PJ, Sudhakar D, Jung IB, Oh BT (2015). Potential use of *Pseudomonas koreensis* AGB-1 in association with *Miscanthus sinensis* to remediate heavy metal (loid)-contaminated mining site soil. J Environ Manag.

[26] Selvakumar G, Kim K, Shagol CC, Joe MM, Sa T (2017). Spore associated bacteria of arbuscular mycorrhizal fungi improve maize tolerance to salinity by reducing ethylene stress level. Plant Growth Regul.

[27] Battini F, Grønlund M, Agnolucci M, Giovannetti M, Jakobsen I (2017). Facilitation of phosphorus uptake in maize plants by mycorrhizosphere bacteria. Sci Rep.

[28] Maathuis FJM, Amtmann A (1999). K^+^ nutrition and Na^+^ toxicity: the basis of cellular K^+^/Na^+^ ratios. Ann Bot.

[29] Hammer EC, Nasr H, Pallon J, Olsson PA, Wallander H (2011). Elemental composition of arbuscular mycorrhizal fungi at high salinity. Mycorrhiza.

[30] Munns R (2005). Genes and salt tolerance: bringing them together. New Phytol.

[31] Shi H, Quintero FJ, Pardo JM (2002). Zhu and JK. The putative plasma membrane Na^+^/H^+^ antiporter SOS1 controls long-distance Na^+^ transport in plants. Plant Cell.

[32] Ji H, Pardo JM, Batelli G, Van Oosten MJ, Bressan RA, Li X (2013). The salt overly sensitive (SOS) pathway: established and emerging roles. Mol Plant.

[33] Gaymard F, Pilot G, Lacombe B, Bouchez D, Bruneau D, Boucherez J, Michaux-Ferrière N, Thibaud JB, Sentenac H (1998). Identification and disruption of a plant shaker-like outward channel involved in K^+^ release into the xylem sap. Cell.

[34] Marten I, Hoth S, Deeken R, Ache P, Ketchum KA, Hoshi T, Hedrich R (1999). AKT3, a phloem-localized K^+^ channel, is blocked by protons. Pro Natl Acad Sci USA.

[35] Selvakumar G, Krishnamoorthy R, Kim K, Sa TM (2016). Genetic diversity and association characters of bacteria isolated from arbuscular mycorrhizal fungal spore walls. PLoS One.

[36] Selvakumar G, Krishnamoorthy R, Kim K, Sa T (2016). Propagation technique of arbuscular mycorrhizal fungi isolated from coastal reclamation land. Eur J Soil Biol.

[37] Xi C, Lambrecht M, Vanderleyden J, Michiels J (1999). Bi-functional *gfp*-and *gusA*-containing mini-Tn5 transposon derivatives for combined gene expression and bacterial localization studies. J Microbiol Methods.

[38] Yim WJ, Kim KY, Lee YW, Sundaram SP, Lee Y, Sa TM (2014). Real time expression of ACC oxidase and PR-protein genes mediated by *Methylobacterium* spp. in tomato plants challenged with *Xanthomonas campestris* pv. *vesicatoria*. J Plant Physiol.

[39] Götz M, Gomes NCM, Dratwinski A, Costa R, Berg G, Peixoto R (2006). Survival of *gfp*-tagged antagonistic bacteria in the rhizosphere of tomato plants and their effects on the indigenous bacterial community. FEMS Microbiol Ecol.

[40] Sheng M, Tang M, Zhang F, Huang Y (2011). Influence of arbuscular mycorrhiza on organic solutes in maize leaves under salt stress. Mycorrhiza.

[41] Bates LS, Waldren RP, Teare ID (1973). Rapid determination of free proline for water-stress studies. Plant Soil.

[42] Phillips JM, Hayman DS (1970). Improved procedures for clearing roots and staining parasitic and vesicular-arbuscular mycorrhizal fungi for rapid assessment of infection. Trans Br Mycol Soc.

[43] Trouvelot A, Kough JL, Gianinazzi-Pearson V, Gianinazzi-Pearson V, Gianinazzi S (1986). Mesure du taux de mycorrhization VA dun systeme radiculaire. Recherche de methods destimation ayant une signification fonctionnelle. Pysiological and genetical aspects of mycorrhizae.

[44] Gerdemann JW, Nicolson TH (1963). Spores of mycorrhizal Endogone species extracted from soil by wet sieving and decanting. Trans Br Mycol Soc.

[45] Miransari M (2011). Interactions between arbuscular mycorrhizal fungi and soil bacteria. Appl Microbiol Biotechnol.

[46] Berta G, Copetta A, Gamalero E, Bona E, Cesaro P, Scarafoni A (2014). Maize development and grain quality are differentially affected by mycorrhizal fungi and a growth-promoting pseudomonad in the field. Mycorrhiza.

[47] Colla G, Rouphael Y, Cardarelli M, Tullio M, Rivera CM, Rea E (2008). Alleviation of salt stress by arbuscular mycorrhizal in zucchini plants grown at low and high phosphorus concentration. Biol Fertil Soils.

[48] Hajiboland R, Aliasgharzadeh N, Laiegh SF, Poschenrieder C (2010). Colonization with arbuscular mycorrhizal fungi improves salinity tolerance of tomato (*Solanum lycopersicum* L.) plants. Plant Soil.

[49] Daei G, Ardekani MR, Rejali F, Teimuri S, Miransari M (2009). Alleviation of salinity stress on wheat yield, yield components, and nutrient uptake using arbuscular mycorrhizal fungi under field conditions. J Plant Physiol.

[50] Abdel Latef AAH, Chaoxing H (2011). Effect of arbuscular mycorrhizal fungi on growth, mineral nutrition, antioxidant enzymes activity and fruit yield of tomato grown under salinity stress. Sci Hortic.

[51] Kishor PBK, Hong ZL, Miao GH, Hu CAA, Verma DPS (1995). Overexpression of Delta-Pyrroline-5-carboxylate Synthetase increases proline production and *Confers Osmotolerance* in transgenic plants. Plant Physiol.

[52] Evelin H, Giri B, Kapoor R (2012). Contribution of *Glomus intraradices* inoculation to nutrient acquisition and mitigation of ionic imbalance in NaCl-stressed *Trigonella foenum-graecum*. Mycorrhiza.

[53] Hashem A, Abd-Allah EF, Alqarawi AA, Al-Huqail AA, Wirth S, Egamberdieva D (2016). The interaction between arbuscular mycorrhizal fungi and endophytic bacteria enhances plant growth of *Acacia gerrardii* under salt stress. Front Microbiol.

[54] Munns R, Tester M (2008). Mechanisms of salinity tolerance. Annu Rev Plant Biol.

[55] Liu FP, Liu HQ, Zhou HL, Dong ZG, Bai XH, Bai P (2014). Isolation and characterization of phosphate-solubilizing bacteria from betel nut (*Areca catechu*) and their effects on plant growth and phosphorus mobilization in tropical soils. Biol Fertil Soils.

[56] Yang CW, Xu HH, Wang LL, Liu J, Shi DC, Wang DL (2009). Comparative effects of salt-stress and alkali-stress on the growth, photosynthesis, solute accumulation, and ion balance of barley plants. Photosynthetica.

[57] Ruiz-Lozano JM, Porcel R, Azcon C, Azcon R (2012). Regulation by arbuscular mycorrhizae of the integrated physiological response to salinity in plants: new challenges in physiological and molecular studies. J Exp Bot.

[58] Giri B, Kapoor R, Mukerji KG (2007). Improved tolerance of *Acacia nilotica* to salt stress by arbuscular mycorrhiza, *Glomus fasciculatum* may be partly related to elevated K/Na ratios in root and shoot tissues. Microb Ecol.

[59] Cantrell IC, Linderman RG (2001). Preinoculation of lettuce and onion with VA mycorrhizal fungi reduces deleterious effects of soil salinity. Plant Soil.

[60] Hamamoto S, Horie T, Hauser F, Deinlein U, Schroeder JI, Uozumi N (2015). HKT transporters mediate salt stress resistance in plants: from structure and function to the field. Curr Opin Biotechnol.

[61] Elhindi KM, El-Din AS, Elgorban AM (2017). The impact of arbuscular mycorrhizal fungi in mitigating salt-induced adverse effects in sweet basil (*Ocimum basilicum* L.). Saudi J Biol Sci.

[62] Ahmad M, Zahir ZA, Asghar HN, Arshad M (2012). The combined application of rhizobial strains and plant growth promoting rhizobacteria improves growth and productivity of mung bean (*Vigna radiata* L.) under salt-stressed conditions. Ann Microbiol.

[63] Younesi O, Moradi A (2014). Effects of plant growth-promoting rhizobacterium (PGPR) and arbuscular mycorrhizal fungus (AMF) on antioxidant enzyme activities in salt-stressed bean (*Phaseolus vulgaris* l.). Agriculture.

[64] Venema K, Quintero FJ, Pardo JM, Donaire JP (2002). The Arabidopsis Na^+^/H^+^ exchanger AtNHX1 catalyzes low affinity Na^+^ and K^+^ transport in reconstituted liposomes. J Biol Chem.

[65] Shabala S, Cuin TA (2008). Potassium transport and plant salt tolerance. Physiol Plant.

[66] Kost T, Stopnisek N, Agnoli K, Eberl L, Weisskopf L (2014). Oxalotrophy, a widespread trait of plant-associated *Burkholderia* species, is involved in successful root colonization of lupin and maize by *Burkholderia phytofirmans*. Front Microbiol.

[67] Kwon SW, Kim JS, Park IC, Yoon SH, Park DH, Lim CK (2003). *Pseudomonas koreensis* sp. nov., *Pseudomonas umsongensis* sp. nov. and *Pseudomonas jinjuensis* sp. nov., novel species from farm soils in Korea. Int J Syst Evol Microbiol.

[68] Toribio J, Escalante AE, Caballero-Mellado J, González-González A, Zavala S, Souza V (2011). Characterization of a novel biosurfactant producing *Pseudomonas koreensis* lineage that is endemic to Cuatro Ciénegas Basin. Syst Appl Microbiol.

[69] Rashid S, Charles TC, Glick BR (2012). Isolation and characterization of new plant growth-promoting bacterial endophytes. Appl Soil Ecol.

[70] Roesti D, Ineichen K, Braissant O, Redecker D, Wiemken A, Aragno M (2005). Bacteria associated with spores of the arbuscular mycorrhizal fungi *glomus geosporum* and *glomus constrictum*. Appl Environ Microbiol.

[71] Long L, Zhu H, Yao Q, Ai Y (2008). Analysis of bacterial communities associated with spores of *Gigaspora margarita* and *Gigaspora rosea*. Plant Soil.

[72] Agnolucci M, Battini F, Cristani C, Giovannetti M (2015). Diverse bacterial communities are recruited on spores of different arbuscular mycorrhizal fungal isolates. Biol Fertil Soils.

[73] Battini F, Cristani C, Giovannetti M, Agnolucci M (2016). Multifunctionality and diversity of culturable bacterial communities strictly associated with spores of the plant beneficial symbiont *Rhizophagus intraradices*. Microbiol Res.

[74] Figurski DH, Helinski DR (1979). Replication of an origin-containing derivative of plasmid RK2 dependent on a plasmid function provided in trans. Proc Natl Acad Sci.

