# Supplementary figures and images for: Spore associated bacteria regulates maize root K+/Na+ ion homeostasis to promote salinity tolerance during arbuscular mycorrhizal symbiosis
Source: BMC Plant Biol. 2018 Jun 5;18:109. doi: 10.1186/s12870-018-1317-2 (PMC5989414; doi:10.1186/s12870-018-1317-2)

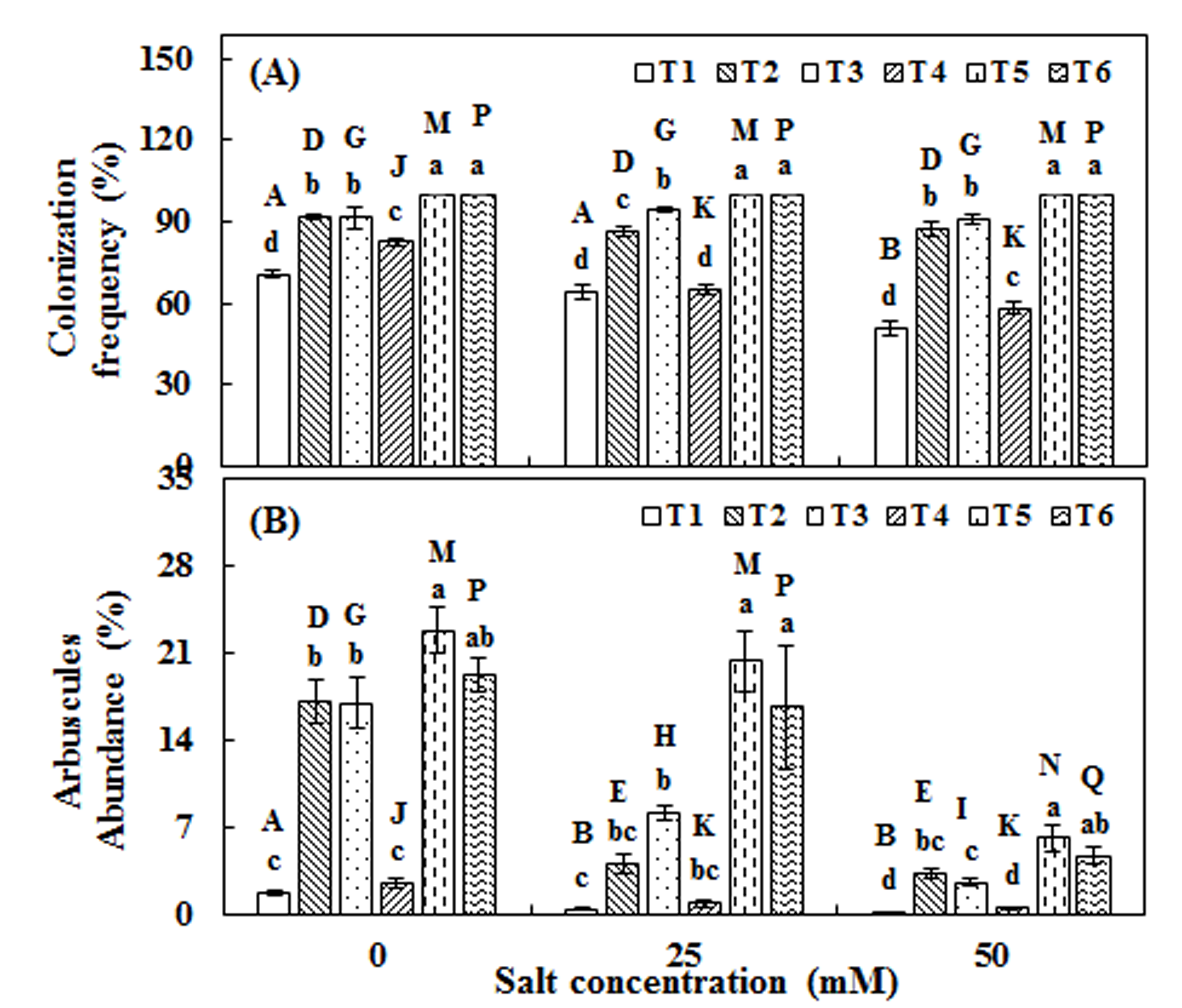

Supplement: Supplementary file 1 — Figure S1. AMF and SAB co-inoculation effect on mycorrhizal colonization frequency and arbuscules abundance. (A) Colonization frequency (B) Arbuscules abundance. T1 – control, T2 - Gigaspora margarita S-23, T3 – Claroideoglomus lamellosum S-11, T4 – Pseudomonas koreensis S2CB35, T5 – T2 + T4, T6 – T3 + T4. Plants were subjected to 0 (0.5 dS/m), 25 (2.5 dS/m) or 50 mM NaCl (4.5 dS/m). Different letters indicate significant differences (P < 0.05) among the treatments at each salt level (a, b, c, d, e, f) or among salt levels for each treatment: T1 (A, B, C), T2 (D, E, F), T3 (G, H, I), T4 (J, K, L), T5 (M, N, O) or T6 (P, Q, R). Each value represents the mean of four replicates ± standard error (SE). (TIF 5431 kb) [file 12870_2018_1317_MOESM1_ESM.tif]

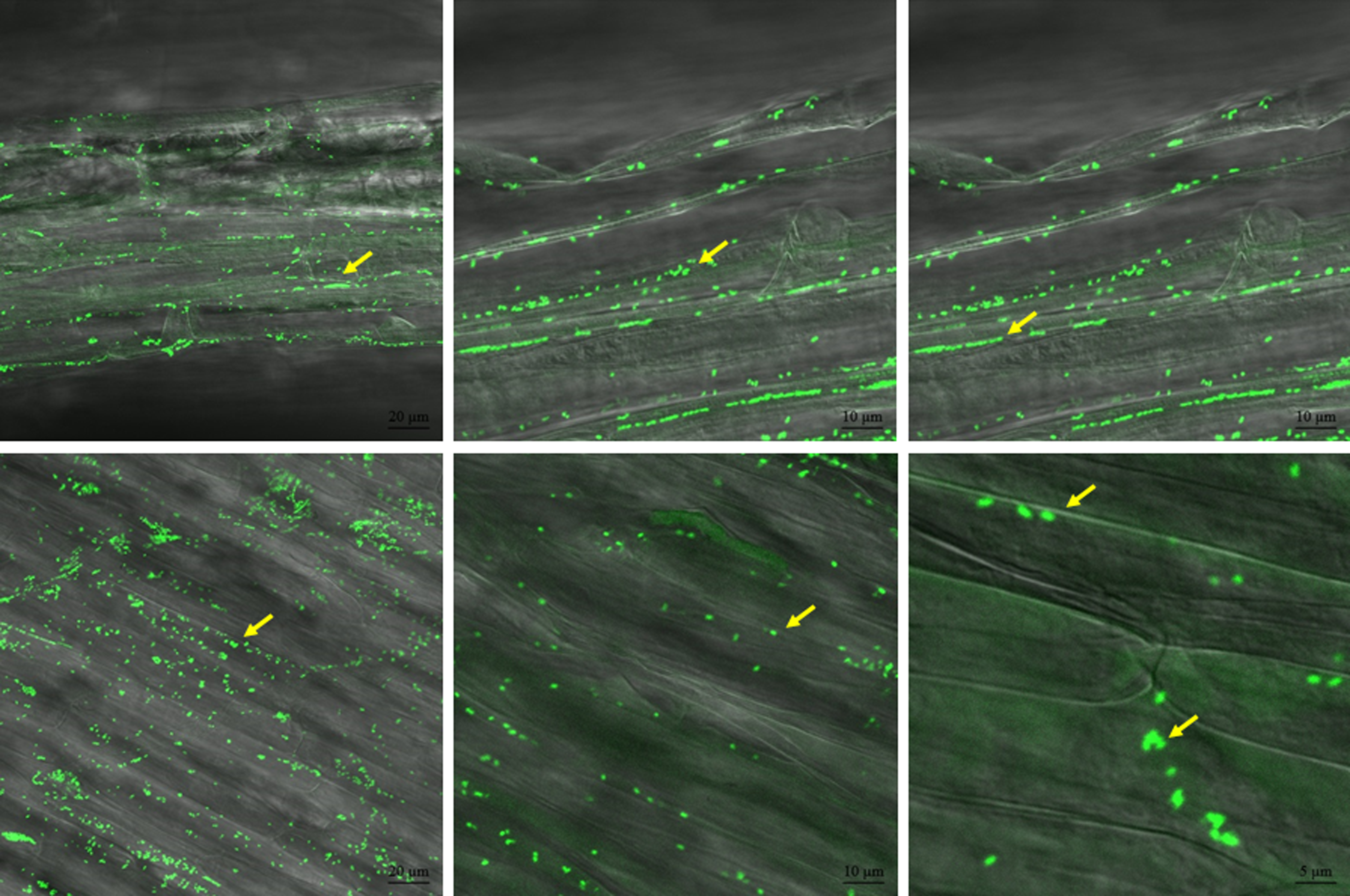

Supplement: Supplementary file 2 — Figure S2. SAB Pseudomonas koreensis S2CB35-gfp colonization in maize plant roots. Arrow indicates the gfp-tagged SAB. (TIF 15878 kb) [file 12870_2018_1317_MOESM2_ESM.tif]

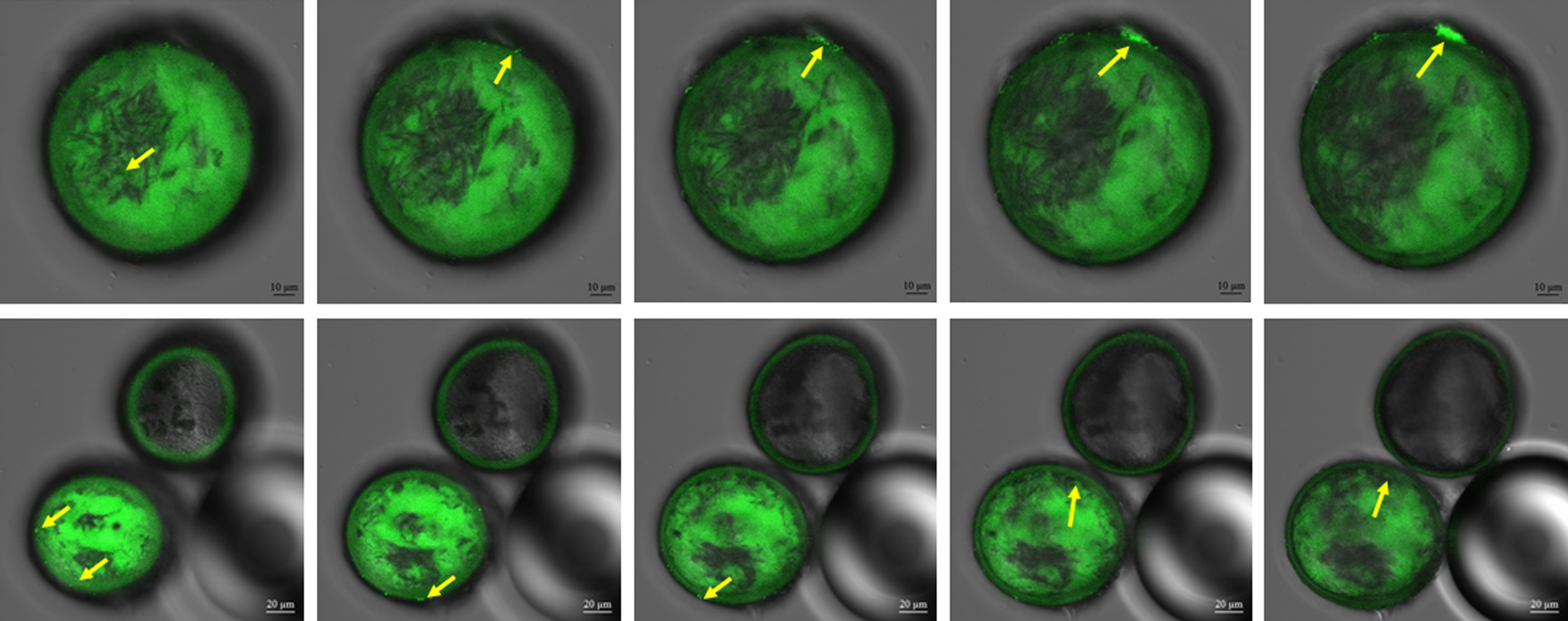

Supplement: Supplementary file 3 — Figure S3. SAB Pseudomonas koreensis S2CB35-gfp colonization on Claroideoglomus lamellosum S-11. Arrow indicates the gfp-tagged SAB. (TIF 14024 kb) [file 12870_2018_1317_MOESM3_ESM.tif]

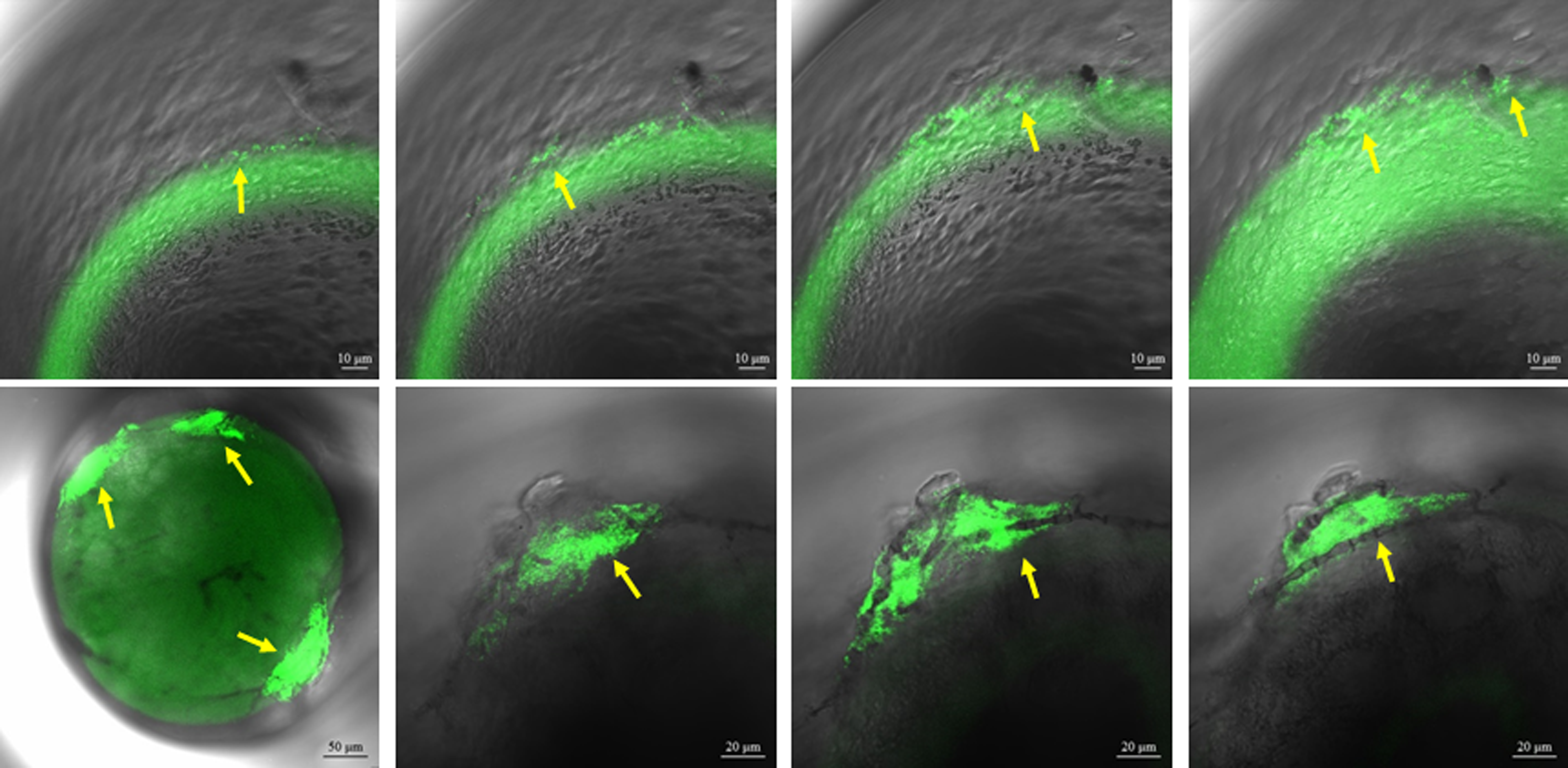

Supplement: Supplementary file 4 — Figure S4. SAB Pseudomonas koreensis S2CB35-gfp colonization on Gigaspora margarita S-23. Arrow indicates the gfp-tagged SAB. (TIF 9889 kb) [file 12870_2018_1317_MOESM4_ESM.tif]
